# MANAGEMENT OF ENDOCRINE DISEASE: Residual adrenal function in Addison’s disease

**DOI:** 10.1530/EJE-20-0894

**Published:** 2020-12-08

**Authors:** Simon H S Pearce, Earn H Gan, Catherine Napier

**Affiliations:** 1 Translational and Clinical Research Institute, Newcastle University, Newcastle, UK; 2 Endocrine Unit, Royal Victoria Infirmary, Newcastle upon Tyne Hospitals NHS Foundation Trust, Newcastle, UK

## Abstract

Over the last 10 years, evidence has accumulated that autoimmune Addison’s disease (AAD) is a heterogeneous disease. Residual adrenal function, characterised by persistent secretion of cortisol, other glucocorticoids and mineralocorticoids is present in around 30% of patients with established AAD, and appears commoner in men. This persistent steroidogenesis is present in some patients with AAD for more than 20 years, but it is commoner in people with shorter disease duration. The clinical significance of residual adrenal function is not fully clear at the moment, but as it signifies an intact adrenocortical stem cell population, it opens up the possibility of regeneration of adrenal steroidogenesis and improvement in adrenal failure for some patients.

## Introduction

Autoimmune Addison’s disease (AAD) is one of the least common autoimmune endocrine conditions, affecting around 1 in 8000 individuals of European ancestry, with a moderate predilection for women (F:M ratio 1.5:1) (1, 2, 3). It is characterised by progressive destruction of the steroid-secreting cells of the adrenal cortex, driven in around 85% of cases by an immune response targeted at steroid 21-hydroxylase, and other steroidogenic enzymes (4, 5). The onset of steroidogenic failure in AAD may be slow. An initial asymptomatic phase is characterised by autoimmune destruction of cells in both adrenal cortices but no hormonal deficit; this is followed by a period of compensated adrenal failure in which circulating renin and ACTH are elevated but with normal circulating steroid hormones, and a potential increase in skin and mucous membrane pigmentation; and latterly by a symptomatic phase of reduced basal and stimulated corticosteroid secretion (6, 7).

There are several notable clinical points about the evolution of disease. First, AAD may have a prolonged compensated phase, with one case report demonstrating a gap of 9 years from the onset of pigmentation, indicating elevated circulating ACTH, to a diagnosis of AAD (8). Post-mortem studies from the 1930s identified areas of adrenocortical hyperplasia as well as areas of destruction and fibrosis in patients with fatal AAD (9, 10). This leads to the idea that in evolving AAD the autoimmune destruction is balanced by an ACTH-driven compensatory hypertrophy which may lead to this gradual decline in adrenocortical function in some patients (7). Secondly, it is well documented that mineralocorticoid deficiency may proceed glucocorticoid deficiency (11, 12). It has been proposed that the high concentrations of cortisol in the zona fasciculata may protect it, to some degree, from immune attack (6, 7). The normal zona fasciculata may have cortisol concentrations between 10 and 50 µM (13), which would be sufficient to produce anergy or apoptosis of antigen-presenting cells* in vitro*. Mineralocorticoids do not have the same potent immunosuppressive effects, which might leave the zona glomerulosa more vulnerable to immune attack. Lastly, patients entering the latter stages of disease have very high circulating ACTH concentrations, typically for months. This means that when challenged with a synacthen (tetracosactrin) test, there is typically minimal rise (≤10%) in the serum cortisol concentration, as the adrenals are already maximally stimulated by endogenous ACTH secretion.

Historically, it has been assumed that AAD is a homogenous disease with regards adrenal steroidogenic function and that without lifelong glucocorticoid and mineralocorticoid replacement medication the disease is invariably fatal. However, several case reports of spontaneous recovery and long-term steroid-independence in well-documented AAD patients have raised the prospect that the outlook is not the same for everybody with AAD (14, 15, 16). There is increasing evidence to suggest preservation of endogenous adrenal steroidogenesis (hereafter referred to as ‘residual adrenal function’) in some patients, even years after AAD diagnosis, and we review the recent advances in our understanding of this phenomenon in this article.

## What is residual adrenal function?

Current thinking is that the destructive autoimmune process in Addison’s disease progresses to a point of no return where complete adrenocortical steroidogenic failure is inevitable and universal. However, following their observation of a male AAD patient who appeared to spontaneously recover adrenal function following 7 years of conventional steroid replacement (14), Smans and Zelissen studied 27 patients with established AAD for signs of residual adrenocortical function (17). Participants’ regular hydrocortisone and fludrocortisone was stopped for 24 h and substituted for dexamethasone, and the serum cortisol response to synacthen was measured by immunoassay. While none of these patients had a normal cortisol response, 10 (37%) did show detectable serum cortisol concentrations, with one patient having a peak value of 100 nmol/L (3.62 µg/dL), suggesting that low level, residual adrenal function might not be uncommon (17). These studies have recently been extended by three different groups using mass spectrometric steroid metabolome assays in a further 249 individuals with established AAD (Table 1) (18, 19, 20).

**Table 1:** Summary of studies of residual adrenal function (RAF) in AAD.

Reference	Method of steroid assay	AAD patients, *n*	RAF, *n*(%)	Serum cortisol >100 nmol/L*, *n*(%)	Comment
Smans & Zelissen (17)	SCI	27	10 (39%)	1 (4%)	On dexamethasone
Vulto * et al.* (18)	Serum LC-MS	20	10 (50%)	–†	Hydrocortisone not omitted
Napier * et al.* (19)	SCI; urine LC-MS	37	6 (16%)	5 (14%)	36 h without replacement medication
Saevik * et al.* (20)	Serum LC-MS	192	58 (30%)	19 (10%)	18 h without replacement medication
Total	-	276	84 (30%)	25/256 (10%)	

*100 nmol/L cortisol is 3.6 µg/dL; ^†^Endogenous cortisol production could not be assessed as patients took hydrocortisone before serum specimens were obtained.

AAD, autoimmune Addison’s disease; SCI, serum cortisol immunoassay; LC-MS, liquid chromatography–tandem mass spectrometry; RAF, residual adrenal function.

**Figure 1:**
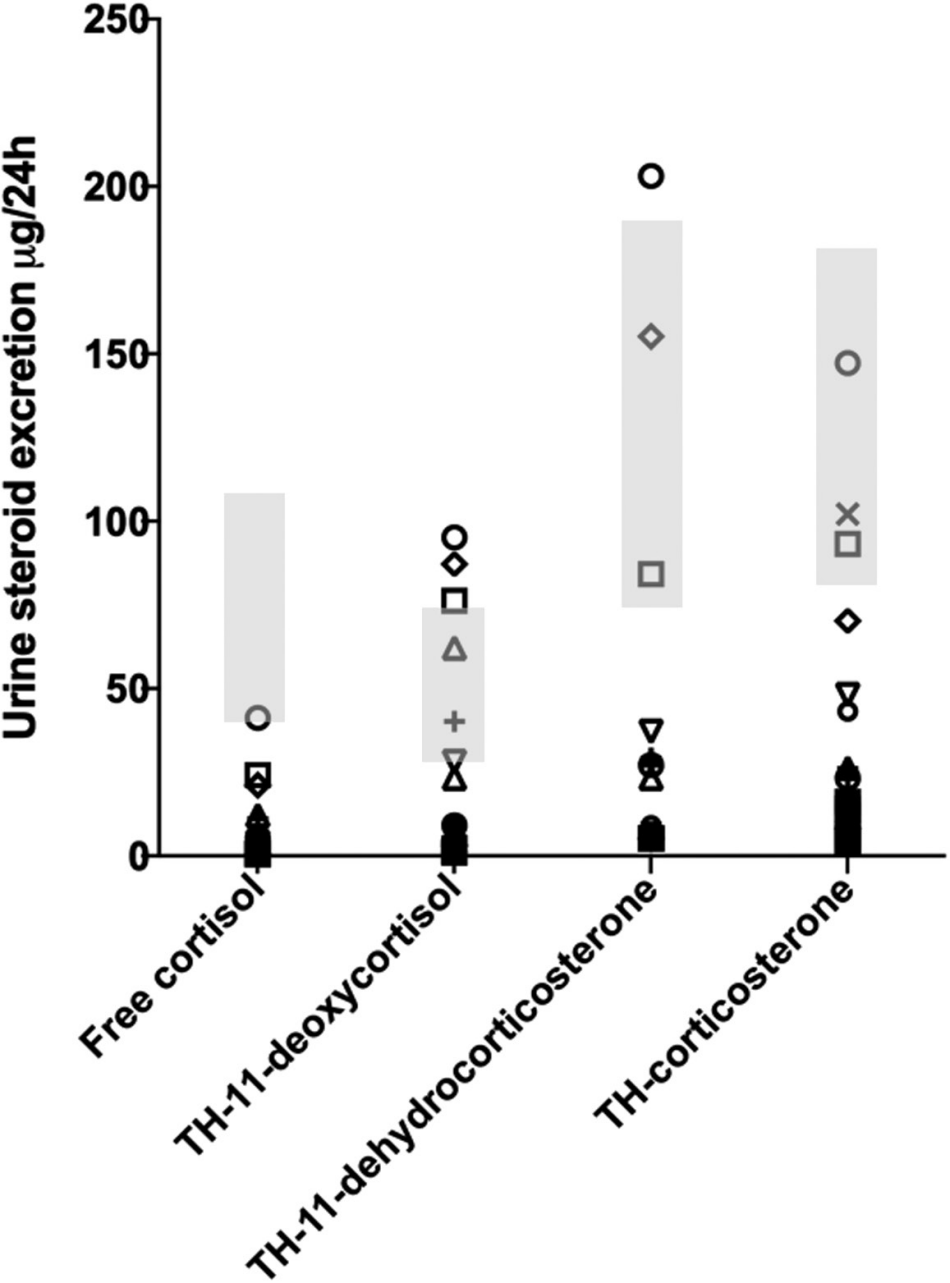
Urine corticosteroid excretion for 37 patients with autoimmune Addison’s disease in a medication-free state (19). Each patient is represented by the same symbol across each metabolite, including the glucocorticoids: free cortisol and tetrahydro-11-deoxycortisol (THS); and the mineralocorticoids: tetrahydro-11-dehydrocorticosterone (THA) and tetrahydro-corticosterone (THB). Grey bars represent interquartile ranges from healthy individuals.

In summary, these three additional studies have used more sensitive assays to establish beyond doubt that AAD patients are heterogeneous in terms of adrenal function, with around 30% having some low level, residual adrenal steroidogenesis for many years following diagnosis. In the two largest studies around 10% had detectable serum cortisol concentration ≥100 nmol/L and 5% had concentrations ≥150 nmol/L (19, 20). Residual adrenal function comprised not just glucocorticoid secretion, but residual mineralocorticoid secretion was also found in many patients (Fig. 1), as might be predicted by the known sequence of adrenocortical cell migration (see subsequently). In contrast, preserved adrenal androgen secretion was not frequent, and indeed because of preserved gonadal steroid secretion, was technically more difficult to evaluate. All studies found that the glucocorticoid precursor 11-deoxycortisol, either in serum or urine, correlated strongly with serum cortisol concentration, making it a potentially useful marker of residual adrenal steroidogenesis that might be ascertained in patients who continued to take their regular medications (18, 19, 20). Interestingly, 6 of 84 participants with residual function had AAD for 20 or more years (19, 20), suggesting that it is a durable state in some patients, although overall residual steroidogenesis was commoner in patients with shorter disease duration. One patient had originally been studied in 2012, 4 years after presentation with AAD when she had a peak stimulated serum cortisol of 184 nmol/L (6.7 µg/dL); repeat measurement in 2016 showed a concentration of 252 nmol/L (9.1 µg/dL) (19, 21). Another notable finding was that residual adrenal function was found in more than 40% (33 of 76) of men with AAD in the largest study (20), whereas it was found in just 18% (2 of 11) in another (19) (the third study did not specify). Lastly, residual adrenal function did not correlate with lack of adrenal crisis or improved quality of life (20).

## Adrenocortical plasticity and adrenal cell turnover

For more than 80 years, it has been known that subcapsular enucleation of the adrenal gland in rodents results in regeneration of adrenal mass in 4–6 weeks (22, 23, 24), with detectable steroidogenic function as early as 8 days following enucleation in rats (22, 23, 24). Early on, administration of exogenous glucocorticoid following adrenal enucleation was observed to inhibit adrenocortical regrowth and to suppress steroidogenesis (22). This was the first indication that both adrenal size and steroidogenic function are primarily maintained by secretion of ACTH from the pituitary gland. During chronic exogenous steroid therapy in man (e.g. prednisolone), ACTH is suppressed leading to adrenal atrophy and functional adrenal failure if exogenous steroids are withdrawn rapidly. In a complementary fashion, excessive pituitary ACTH secretion during Cushing’s disease leads to adrenal gland hyperplasia, as well as hypersecretion of glucocorticoid. Similarly, in the inherited defects of the steroidogenic enzymes, the high ACTH levels promote adrenal growth: a series of conditions collectively termed congenital adrenal hyperplasia.

Knowledge about the cell biology of adrenal zonation and steroidogenesis in human is imperfect, largely because there are significant differences between human and rodent adrenal morphology and secretory function that preclude confident cross-species extrapolations. Nevertheless, the widely accepted model suggests that adrenocortical stem cells (ACSC), which have a subcapsular location, proliferate and differentiate into cells that have the ability to manufacture and secrete steroid hormones under the influence of ACTH (25, 26). Following proliferation, the daughter cells migrate centripetally while undergoing a zone-specific differentiation programme (26). First they transit through the zona glomerulosa, taking on an aldosterone secreting phenotype. Then into the zona fasciculata where they secrete cortisol and finally they end up in the innermost cortical zone, the zona reticularis where they secrete adrenal androgens. There is a terminal apoptotic layer at the border between the zona reticularis and the adrenal medulla. Cell migration, studied with thymidine autoradiography, suggest that the passage from the subcapsular region to apoptotic layer takes about 100 days in the rat (27). However, recent cell tracking studies using transgenic murine models suggests sex-specific rates of adrenocortical turnover, with a repopulation time of 9 months in adult males compared to just 3 months in female mice (28). Interestingly, similar finding have been made in neonatal mice (29) and there appears to be an androgen-dependent inhibition of stem cell proliferation in both these models. These animal studies form a framework to understand more about human adrenocortical plasticity. Furthermore, the sex differences in adrenocortical turnover may also be relevant to the higher prevalence of residual adrenal function found in men with AAD.

## Residual adrenal function in context of autoimmunity

Numerous autoimmune conditions exhibit waxing and waning disease activity, usually referred to as a relapsing and remitting course, typified by Graves’ hyperthyroidism or multiple sclerosis (30). This contrasts to type 1 diabetes (T1D), in which the autoimmune destruction appears persistent and relentless. Nevertheless, persistent insulin secretion, characterised by differing C-peptide concentrations endures in many individuals (31). Interestingly, a recent large study of Scottish patients with T1D found that persistent C-peptide secretion was also commoner in males (32), paralleling the findings in AAD (28).

It has been largely assumed that autoimmunity in AAD eventually leads to the destruction of all adrenocortical function (6). However, these recent findings demonstrate that complete destruction of steroidogenic function does not occur in a substantial minority of AAD patients. This could reflect that the autoimmune process itself may remit over time (6, 11). In addition, residual adrenal function persisting for many years must be underpinned by intact ACSC function and we speculate that these cells may be spared autoimmune destruction as they do not express steroid 21-hydroxylase, or other steroidogenic enzymes which are the target of the immune attack (4, 33, 34). A durable, albeit perhaps reduced, ACSC population has the potential to proliferate indefinitely and repopulate the adrenal cortex, particularly if the autoimmune process is checked by immunomodulatory medication or remits naturally. This possibility stimulated us to perform a series of small experimental medicine studies to try and improve steroidogenic function in AAD (Table 2) (21, 35, 36).

**Table 2:** Summary of interventional studies in AAD.

Reference	Cohort	Intervention	AAD patients, *n*	Outcome	Comment
Pearce * et al.* (35)	Newly diagnosed AAD	Rituximab	6	1 patient (17%) steroid-free at 1 year	Peak serum cortisol 434 nmol/L; Adrenal failure returned
Napier * et al.* (36)	Newly diagnosed AAD	Rituximab and Synacthen	13	4 patients (30%) with peak serum cortisol ≥99 nmol/L at 18 month	No patient was able to stop replacement medication
Gan *et al*. (21)	Established AAD	Synacthen	13	2 patients (15%) became medication free	One patient remained steroid independent for 8 years

## Could residual adrenal function be exploited to cure AAD?

Most patients presenting for the first time with AAD have circulating cortisol concentrations that, despite being abnormal, are easily detectable with conventional immunoassays. We found a median serum cortisol of 125 nmol/L (4.5 µg/dL) in twenty individuals at the time of AAD presentation, with only three of 20 having levels below an arbitrary 24 nmol/L (0.9 µg/dL) limit of quantitation (19). This clearly demonstrated that patients with AAD have ongoing steroidogenesis at the time of diagnosis and we wished to explore whether this could be rescued. We treated 13 newly diagnosed AAD patients using a combination of high dose depot synacthen, in order to maintain trophic stimulation of steroidogenesis, and the B lymphocyte depleting agent rituximab in an attempt to combat the immune attack (36). No patient fully recovered adrenal function but 50% of participants had some improvement in steroidogenic function, leaving 4 (30%) including 2 men, with detectable serum cortisol of 99 nmol/L (3.6 µg/dL) or more, after 18 months (Table 2) (36). This rate of residual adrenal function is greater than that found spontaneously in the observational studies, where only 10% of individuals had serum cortisol concentrations ≥100 nmol/L (17, 18, 19, 20).

Could adrenal steroidogenesis be salvageable further into the course of the disease? With this question in mind, we administered high dose synacthen to 13 unselected patients with AAD established for more than a year (21). Two patients who both had a residual adrenal function, with baseline serum cortisols of 184 and 219 nmol/L, responded by augmenting their steroidogenesis enough to stop replacement medication (21). In one patient the steroidogenesis slowly decreased once synacthen injections were ceased, but the other participant remained off medication for 8 years. Although only a transient benefit was found for one of the patients, this remains the promise of residual adrenal function and additional studies, particularly in those with highest residual steroidogenesis and an insidious onset, are now needed to target this small but important group of AAD patients who might reap great benefit from new approaches. This leads on to quantitative questions about the significance of different degrees of residual adrenal function.

## Can we define useful residual adrenal steroidogenic function?

Patients with T1D and C-peptide concentrations above 200 pmol/L have been demonstrated to have lower rates of microvascular complications and reduced hypoglycaemia (37). However, even those with lesser degrees of endogenous insulin secretion also have improved outcomes, including lower glycosylated haemoglobin, lower insulin requirement and reduced urine albumin excretion (38). Therefore, two key unresolved questions are what defines residual adrenal function; and whether at a certain level residual steroidogenesis might be of clinical benefit for AAD patients? In two of the recent studies, mass spectrometric analysis was able to quantitate serum cortisol down to concentrations of <0.01 and 0.9 nmol/L, respectively (18, 20), whereas, in the third study, urine cortisol was quantified down to 3 nmol/L (19). However, these very low corticosteroid levels may be neither physiologically relevant nor indeed reflect true adrenal cortisol production, as extra-adrenal cortisol generation is reported from many tissues including intestine, thymus, lung, skin and brain (39, 40). In the largest observational studies of AAD patients (19, 20), only around 10% had detectable serum cortisol concentration ≥100 nmol/L, with 5% having concentrations ≥150 nmol/L. This means that although the residual adrenal function was commoner than expected, in most AAD patients the mechanism by which it could be contributing to their health is not obvious at the moment. Moreover, reduced frequency or lack of adrenal crisis might be an obvious possible correlate of residual adrenal function, given that symptoms of adrenal crisis predominantly relate to mineralocorticoid deficiency, there may not be a direct relation between it and residual cortisol secretion. A much larger study is now necessary to find out whether the few patients with residual steroidogenesis in the top centiles of steroid production have some associated health benefit.

## Conclusions

Residual adrenal function, characterised by detectable but low concentrations of cortisol and other corticosteroids, is present in around 30% of AAD patients. However, only around 5–10% have serum cortisol concentrations over 100–150 nmol/L which might potentially be associated with some clinical benefit, although it seems most likely that too few individuals in this category have currently been studied to fully characterise this. Serum or urine 11-deoxycortisol may be a sensitive marker for residual adrenal function. Owing to adrenocortical stem cell plasticity, patients with residual adrenal function are important subjects for further study to see if the promise of a surviving pluripotent stem cell population can be exploited to enhance steroidogenesis and ameliorate adrenocortical failure.

## Declaration of interest

The authors declare that there is no conflict of interest that could be perceived as prejudicing the impartiality of this review.

## Funding

Original work on this subject has been funded by Medical Research Council http://dx.doi.org/10.13039/501100000265 grants MR/J002526/1, G0900001 and G07017632. Additional infrastructure support was made available through the National Institute for Health Research http://dx.doi.org/10.13039/100005622 (NIHR) Newcastle Biomedical Research Centre based at Newcastle Hospitals NHS Foundation Trust and Newcastle University http://dx.doi.org/10.13039/501100000774, the Newcastle Clinical Research Facility, and Roger and Virginia Robotham. S P has received speaker fees from Sanofi http://dx.doi.org/10.13039/100004339, Quidel and Berlin Chemie, and has consulted for Apitope BV.
